# Evaluation of dosimetric effect of leaf position in a radiation field of an 80‐leaf multileaf collimator fitted to the LINAC head as a tertiary collimator

**DOI:** 10.1120/jacmp.v7i3.2310

**Published:** 2006-08-24

**Authors:** Than S. Kehwar, Anup K. Bhardwaj, Shiv K. Chakarvarti

**Affiliations:** ^1^ Department of Radiation Oncology University of Pittsburgh Cancer Institute Pittsburgh Pennsylvania U.S.A.; ^2^ Department of Radiation Oncology Postgraduate Institute of Medical Education and Research Chandigarh India; ^3^ Department of Applied Physics National Institute of Technology Kurukshetra India

**Keywords:** 3D‐CRT, IMRT, multileaf collimator, scatter factors, penumbra width, percentage depth dose

## Abstract

This study evaluates changes in the dosimetric characteristics of a Varian Millennium 80‐leaf multileaf collimator (MLC) in a radiation field. In this study, dose rate, scatter factor, percentage depth dose, surface dose and dose in the buildup region, beam profile, flatness and symmetry, and penumbra width measurements were made for 6‐MV and 15‐MV photon beams. Analysis of widths between 50% dose levels of the beam profiles to reflect the field size at the level of profile measurement shows a significant difference between the fields defined by MLC and/or jaws and MLC (zero gap) and the fields defined by jaws only. The position of the MLC leaves in the radiation field also significantly affects scatter factors. A new relationship has, therefore, been established between the scatter factors and the position of the MLC, which will indeed be useful in the dose calculation for irregular fields. Penumbra widths increase with field size and were higher for fields defined by jaws and/or MLC than jaws and MLC (zero gap) by 1.5 mm to 4.2 mm and 3.8 mm to 5.0 mm, for 6‐MV, and 1.5 mm to 2.4 mm and 3.0 mm to 5.6 mm, for 15‐MV, at 20% to 80% and 10% to 90% levels, respectively. The surface dose and the dose in the buildup region were smaller for fields defined by jaws and MLC (zero gap) than the fields defined by jaws and/or MLC for both photon energies. No significant differences were found in percentage depth dose beyond $d_{\max}$, beam profiles above 80% dose level, and flatness and symmetry for both energies. The results of this study suggest that while one collects linear accelerator beam data with a MLC, the effects of the positions of the MLC leaves play an important role in dosimetric characteristics of 3D conformal radiation therapy as well as intensity‐modulated radiotherapy.

PACS number: 87.53.Dq

## I. INTRODUCTION

Modern linear accelerators equipped with multileaf collimator (MLC) systems provide means to deliver 3D conformal radiation therapy (3D‐CRT)^(^ ^1^ ^–^ ^4^ ^)^ and intensity‐modulated radiation therapy (IMRT).^(^ ^5^ ^–^ ^7^ ^)^ The use of a MLC system for delivering 3D‐CRT and IMRT requires the delivery of complex beam arrangements having multiple, irregularly shaped radiation fields and, therefore, depends on accurate dosimetric parameters of the MLC.^(^ ^8^ ^–^ ^16^ ^)^ In 3D‐CRT, dose and/or monitor units are verified using semi‐empirical methods of dose calculation. In some radiotherapy treatment‐planning systems, the dose calculation and planning algorithms are based on these methods but the methods do not account for the changes in dosimetric parameters due to the positions of the MLC leaves in the radiation field. The influence of the MLC leaves on dosimetric parameters depends on the type of MLC system and its placement in the treatment head.

In Varian linear accelerators, the MLC system is used as a tertiary collimator, that is, below the collimator jaws, which influences dosimetric data in a way entirely different than in other MLC systems. The dosimetric data obtained at the time of acceptance testing and commissioning of a linear accelerator do not account for the influence of MLC leaves. Hence, any change in dosimetric characteristics of beam data may lead to an error in the dose calculations either done by computer or manually. In some dose computation algorithms for tertiary collimator systems (MLC systems) for irregular fields, similar dosimetric and scatter quantities are used as for Co‐60 teletherapy irregular fields,^(17)^ that is, only collimator scatter and phantom scatter factors are used, which may cause significant differences in computed and actual dose delivery. Hence, the influence of the MLC system must be taken into account.

In this paper, an evaluation of the performance and dosimetric characteristics of the field‐defining systems, such as MLC and collimator systems, is presented. The dosimetric characteristics include dose rates, scatter factors, central axis percentage depth dose, surface dose, dose in buildup region, beam profiles, flatness and symmetry, and penumbra width for 6‐MV and 15‐MV photon beams, for the fields defined by jaws only (MLC in park position), MLC only (fixed jaws at $35\times 35\;{\text{cm}}^{2}$), and jaws with MLC (with zero gap).

## II. METHODS AND MATERIALS

Dosimetric characteristics of the Varian linear accelerator, CLINAC DHX (2300 CD), with Millennium 80‐leaf MLC system, were measured for 6‐MV and 15‐MV photon energies.

### A. Millennium 80 MLC system

The Varian Millennium 80 MLC system consists of a MLC head assembly and control system. The MLC system is attached to the head of the CLINAC DHX (2300 CD) as a tertiary collimator consisting of 40 opposed leaf pairs of tungsten. The leaves are mounted in two leaf banks below X‐jaws. The leaf width, at the isocenter level, of the first and fortieth leaf pairs is 1.4 cm; all others are 1.0 cm. The MLC leaves travel on a carriage to extend their movement across the field. The effective shielding length of the leaves at the isocenter level is 15 cm. Hence, the maximum leaf extension beyond the most retracted leaf on the same side can only be 15 cm. Extension of the leaves out to the field center is not possible when large fields are used, and it is a most severe limitation for large field widths. In some situations, the entire carriage can move so that the leaves can extend over the field center.

### B. Beam data acquisition

#### B.1 Measurement conditions

Measurements were made in air and in water for the following field‐defining methods:

1. fields defined by collimator X and Y jaws only (jaws only) when MLC leaves are in park position
2. fields defined by MLC leaves only (MLC only) for fixed collimator jaws opening at $35\times 35\;{\text{cm}}^{2}$
3. fields defined by collimator jaws and MLC leaves with perfect matching of the leaf end and jaw, that is, simultaneous tracking of collimator jaws and MLC leaves with their ends matching (zero gap)

#### B.2 Dose rate and scatter factors

The output data were measured in air and in water using a 0.6 cm^3^ PTW (PTW‐Freiburg) waterproof ion chamber and a PTW UNIDOS digital electrometer. Measurements were made in a WP 1D (Scanditronix Wellhofer) water phantom. The WP 1D phantom consists of a cubic tank with inner dimensions of 34 cm×40 cm×35 cm (width×length×depth) and a 1D motor‐driven servo, which is used to position the 0.6 cm^3^ PTW ion chamber in both mediums, with positional accuracy of ±0.4 mm and reproducibility of ±0.1 mm. All measurements were made in source‐to‐axis (SAD) setup (SAD=100 cm) for square fields ranging from $4\times 4\;{\text{cm}}^{2}$ to $30\times 30\;{\text{cm}}^{2}$. For in air measurements, appropriate buildup caps were fitted to the ion chamber for 6‐MV and 15‐MV photon energies. Vander (PTW‐Freiburg) has provided buildup caps along with ion chambers for 6‐MV and 15‐MV photon energies to take dose measurements in air. In water, output measurements were made at the depth of maximum dose buildup, that is, 1.5 cm and 3.0 cm for 6‐MV and 15‐MV energies, respectively. The measured output data were converted into the dose rate (DR) and scatter factors (SFs).

#### B.3 Percentage depth dose and beam profile

The central axis percentage depth dose (PDD) and beam profile measurements were made using the RFA‐300 (Scanditronix Wellhofer) 3D radiation field analysis system controlled by Omni Pro Accept computer software. The RFA‐300 consists of a cubic water tank with inner dimensions of 49.5 cm×49.5 cm×49.5 cm (width×length×depth). The drive mechanism of the scanning system has a positional accuracy of ±0.5 mm and reproducibility of ±0.1 mm. Silicon semiconductor diode (*p*‐type) detectors (Scanditronix Wellhofer), with a diameter of 2.5 mm, designed for photon beam measurements, were used. For all measurements, the field detector was positioned with the help of laser lights such that the front face of the detector was normal to the beam direction and parallel to the water surface. A reference detector was placed in one quadrant of the radiation field such that it did not interfere with the readings of the field detector. The sampling resolution and Δ dose (a finite variation in readings) were set to 0.2 mm and 2%, respectively.

The PDD and beam profile were measured in source‐to‐surface (SSD) setup, that is, SDD=100 cm, for both photon beams for all field‐defining methods described in section B.1(1), (2), and (3). The PDD measurements were made for square fields of $4\times 4\;{\text{cm}}^{2}$, $10\times 10\;{\text{cm}}^{2}$, and $20\times 20\;{\text{cm}}^{2}$ and for the depths ranging from 0 cm to 30 cm. Cross‐plane beam profile measurements were done at maximum dose buildup $\left(d_{\max}\right)$ and 10 cm depth for the $4\times 4\;{\text{cm}}^{2}$, $10\times 10\;{\text{cm}}^{2}$, $20\times 20\;{\text{cm}}^{2}$, and $30\times 30\;{\text{cm}}^{2}$ fields. Scanning dimensions for beam profile measurements were taken with an additional margin of 10 cm on both the sides for fields smaller than $20\times 20\;{\text{cm}}^{2}$ and a margin of 5 cm for fields greater than $20\times 20\;{\text{cm}}^{2}$.

## III. RESULTS AND DISCUSSION

### A. Dose rate and scatter factors

**Figure 1:**
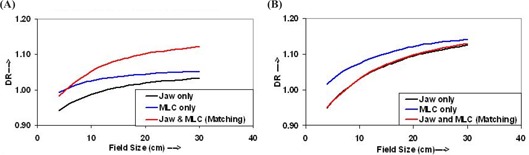
Dose rates (DR) for the 6‐MV photon beam (a) in air and (b) in water for field‐defining methods described in section B.1(1), (2), and (3)

**Figure 2:**
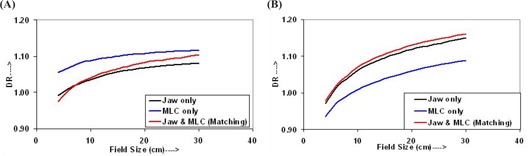
Dose rates (DR) for the 15‐MV photon beam (a) in air and (b) in water for field sizes defined by jaw only, MLC only, and jaw and MLC (matching)

For DR calculations, dose conversion factors were determined for the ion chamber with respect to the absolute dose at reference field size and reference depth for each photon energy. For both photon energies and all field sizes, DR in air, that is, dose per monitor unit (cGy/MU), and in water was calculated from output data using the above dose conversion factors, and are shown in Figs. 1 and 2 for 6‐MV and 15‐MV photon beams, respectively. Data shown in Figs. 1(a) and 2(a) are for DR in air, and those shown in Figs. 1(b) and 2(b) are for DR in water, for 6‐MV and 15‐MV photon beams, respectively.

The increase in the DR as shown in Fig. 1(a) is more pronounced for the fields defined by jaws with a MLC and have higher values compared to the fields defined by jaws only and/or MLC only. However, for field sizes smaller than $5\times 5\;{\text{cm}}^{2}$, the DRs for field sizes defined by jaws with a MLC are smaller than those defined by a MLC only. Figure 1(b) shows that, for the 6‐MV photon beam, the DR (in water) for field sizes defined by the MLC only is higher than that defined by the jaws only and/or jaws with a MLC. On the other hand, the DRs for the field sizes defined by jaws only and jaws with MLC are very similar, that is, within 0.4%.

Figure 2(a) shows curves of the DR in air for the 15‐MV photon beam. The DR of the field sizes defined by a MLC only is higher than that defined by jaws only and/or jaws with a MLC and increases slowly with field size. For the given field size, the differences in the DR for field sizes defined by jaws only and jaws with MLC is also very small; for field sizes greater than $8\times 8\;{\text{cm}}^{2}$, the DR is slightly higher for field sizes defined by jaws with a MLC compared with that defined by jaws only. The DR (in water) for the 15‐MV photon beam, shown in Fig. 2(b), shows that the DR for the fields defined by a MLC only is lower than that defined by jaws only and/or jaws with a MLC, and close to each other for the fields defined by jaw only and jaws with MLC.

The difference in the DR variation in air and in water for both energies is only due to a change in the degree of the scatter radiation from the LINAC collimation and MLC systems and the phantom. The angle of scattering and the energy of the scattered radiation are highly dependent on the photon energy. This is more appreciable for the field sizes less than $5\times 5\;{\text{cm}}^{2}$ and $8\times 8\;{\text{cm}}^{2}$ for 6‐MV and 15‐MV photon energies; hence there are smaller DRs for the fields defined by jaws and MLC (zero gap) than that for the fields defined by jaws only and/or MLC only.

**Figure 3:**
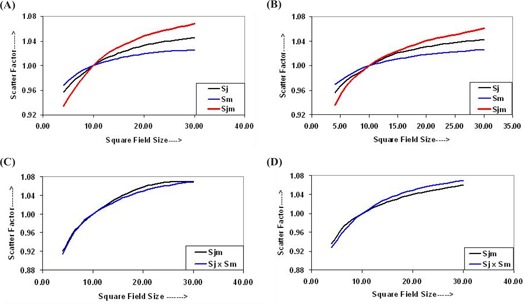
Plot between scatter factors $S_{j},\;S_{m},\;\text{and}\;S_{\text{jm}}$ and field size for (a) the 6‐MV photon beam and (b) the 15‐MV photon beam for air dosimetry. Curves for the product of $S_{j}$ and $S_{m}$, and $S_{\text{jm}}$ for (c) 6‐MV photon beams and (d) 15‐MV photon beams

The output data were normalized with respect to that of the reference field, that is, $10\times 10\;{\text{cm}}^{2}$, to determine respective relative scatter factors (SFs) for each set of measurements. The output data obtained from in air and in water dosimetry give a set of SFs related to jaws only, MLC only, and jaws with MLC fields. These are as follows: for air dosimetry (i) collimator (jaw) scatter factor $\left(S_{j}\right)$, (ii) MLC scatter factor $\left(S_{m}\right)$, and (iii) jaw+MLC scatter factor $\left(S_{\text{jm}}\right)$; and for water dosimetry (iv) collimator (jaw) and phantom (water) scatter factor $\left(S_{\text{jp}}\right)$, (v) MLC and phantom(water) scatter factor $\left(S_{\text{mp}}\right)$, and (vi) jaw+MLC and phantom (water) scatter factor $\left(S_{\text{jmp}}\right)$. The SFs in air, $S_{j},\;S_{m},\;\text{and}\;S_{\text{jm}}$, are shown in Figs. 3(a) and 3(b), respectively, for 6‐MV and 15‐MV photon beams. Figures 3(c) and 3(d), for 6‐MV and 15‐MV photon beams, show that the product of $S_{j}$ and $S_{m}$ is close to $S_{\text{jm}}$, within 1%. In radiation dosimetry, a tolerance level of ±2% is considered fairly accurate. Therefore, there is a relationship between $S_{j},\;S_{m},\;\text{and}\;S_{\text{jm}}$, which can be expressed as follows:

(1) $$S_{\text{jm}}(r)=S_{j}(r)\times S_{m}(r),$$

where *r* is the square field of the radiation beam. Equation (1) shows that $S_{\text{jm}}$ is the product of $S_{j}$ and $S_{m}$. To calculate the DR in air for a field defined by jaws and MLC combination, the following relation can be used:

(2) $${\text{DR}}_{\text{jm}}(r)=\text{DR}(\text{Ref}.\;\text{Field})\times S_{j}(r)\times S_{m}(r),$$

where $\text{Ref}.\;\text{Field}=10\times 10\;{\text{cm}}^{2}$. Equation (2) is used to calculate the DRs for the field defined by jaws with a MLC using the DRs of the $10\times 10\;{\text{cm}}^{2}$ field for all three field‐defining methods and respective values of SFs in air. It is clear from Tables 1and 2 that the DRs for the field defined by jaws with a MLC are very close to the measured DRs with an accuracy of ±1.5% for both 6‐MV and 15‐MV photon energies. Hence, the DR for the fields defined by jaws plus a MLC should be taken as the reference field DR for both photon energies. Equation (2) now can be rewritten as

(3) $${\text{DR}}_{\text{jm}}(r)={\text{DR}}_{\text{jm}}(\text{Ref}.\;\text{Field})\times S_{j}(r)\times S_{m}(r).$$

**Table 1:** Differences in calculated dose rates in air, for the 6‐MV photon beam, with the measured dose rates for different field‐defining methods

Measured DRs	Calculated DRs	% Difference
${\text{DR}}_{\text{jm}}(r)$	${\text{DR}}_{j}(10\times 10)\times S_{j}(r)\times S_{m}(r)$	<±1.5
${\text{DR}}_{\text{jm}}(r)$	${\text{DR}}_{m}(10\times 10)\times S_{j}(r)\times S_{m}(r)$	<±6.3
${\text{DR}}_{\text{jm}}(r)$	${\text{DR}}_{\text{jm}}(10\times 10)\times S_{j}(r)\times S_{m}(r)$	<±1.5

${\text{DR}}_{\text{jm}}(r)=$ measured DR for *r* field size defined by collimator jaw and MLC; ${\text{DR}}_{\text{jm}}(10\times 10)=$ measured DR for $10\times 10\;{\text{cm}}^{2}$ field size defined by jaw and MLC; ${\text{DR}}_{j}(10\times 10)=$ measured DR for $10\times 10\;{\text{cm}}^{2}$ field size defined by jaw only; ${\text{DR}}_{m}(10\times 10)=$ measured DR for $10\times 10\;{\text{cm}}^{2}$ field size defined by MLC only

**Table 2:** Differences in calculated dose rates in air, for the 15‐MV photon beam, with the measured dose rates for different field‐defining methods

Measured DRs	Calculated DRs	% Difference
${\text{DR}}_{\text{jm}}(r)$	${\text{DR}}_{j}(10\times 10)\times S_{j}(r)\times S_{m}(r)$	<±7.2
${\text{DR}}_{\text{jm}}(r)$	${\text{DR}}_{m}(10\times 10)\times S_{j}(r)\times S_{m}(r)$	<±7.5
${\text{DR}}_{\text{jm}}(r)$	${\text{DR}}_{\text{jm}}(10\times 10)\times S_{j}(r)\times S_{m}(r)$	<±1.3

${\text{DR}}_{\text{jm}}(r)=$ measured DR for *r* field size defined by collimator jaw and MLC; ${\text{DR}}_{\text{jm}}(10\times 10)=$ measured DR for $10\times 10\;{\text{cm}}^{2}$ field size defined by jaw and MLC; ${\text{DR}}_{j}(10\times 10)=$ measured DR for $10\times 10\;{\text{cm}}^{2}$ field size defined by jaw only; ${\text{DR}}_{m}(10\times 10)=$ measured DR for $10\times 10\;{\text{cm}}^{2}$ field size defined by MLC only

Equation (3) can also be solved for other jaws and MLC settings.

**Figure 4:**
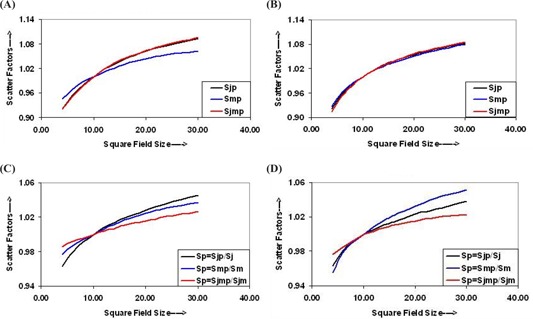
Curves between scatter factors in water and field size for (a) 6‐MV photon beams and (b) 15‐MV photon beams. (c) Phantom scatter factors for 6‐MV photon beams and (d) phantom scatter factors for 15‐MV photon beams determined using scatter factors for field sizes defined by jaw only, MLC only, and jaw and MLC (matching) in air and in water.

The SFs in water, $S_{\text{jp}}$, $S_{\text{mp}}$, and $S_{\text{jmp}}$, are shown in Figs. 4(a) and 4(b), respectively, for 6‐MV and 15‐MV photon beams. The quantitative analysis of 6‐MV photon beam SFs, measured in water, shown in Fig. 4(a), reveals that $S_{\text{jp}}$ and $S_{\text{jmp}}$ are very close (<0.3%), and $S_{\text{mp}}$ is significantly different than the values of $S_{\text{jp}}$ and $S_{\text{jmp}}$. In Fig. 4(b), of 15‐MV SFs in water, the $S_{\text{jp}}$, $S_{\text{mp}}$, and $S_{\text{jmp}}$ do not differ significantly from each other (within ±1%). The values of $S_{p}$ were calculated from $S_{j}$, $S_{m}$, and $S_{\text{jm}}$, and $S_{\text{jp}}$, $S_{\text{mp}}$, and $S_{\text{jmp}}$ for 6‐MV and 15‐MV photon beams, and are shown in Figs. 4(c) and 4(d). Each energy has three sets of $S_{p}$ data, which makes it very difficult to choose the correct set of values for accurate dose/DR calculations. We calculated the DR for all combinations of the values and found that the following relation can be used for these settings:

(4) $${\text{DR}}_{\text{jmp}}(r,\;d)={\text{DR}}_{\text{jmp}}(\text{Ref}.\;\text{Field},\;d_{\text{ref}})\times S_{j}(r)\times S_{m}(r)\times S_{p}(r),$$

where ${\text{DR}}_{\text{jmp}}(r,d)=$ dose rate in water (phantom) for field *r* at depth *d* for jaws plus a MLC combination, ${\text{DR}}_{\text{jmp}}$(Ref. Field, $d_{\text{ref}}$) = dose rate in water (phantom) for reference field size ($10\times 10\;{\text{cm}}^{2}$) at $d_{\text{max}}$, and $S_{p}(r)=$ phantom scatter for jaws plus MLC field.

### B. Percentage depth dose (PDD)

**Figure 5:**
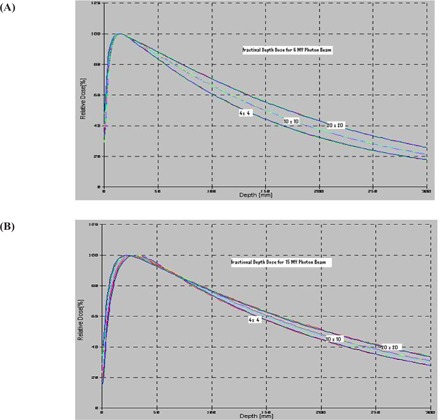
Plots of percentage depth dose (PDD) versus depth for $4\times 4\;{\text{cm}}^{2}$, $10\times 10\;{\text{cm}}^{2}$, and $20\times 20\;{\text{cm}}^{2}$ field sizes defined by jaw only, MLC only, and jaw and MLC (matching) for (a) 6‐MV photon beam and (b) 15‐MV photon beam

The PDD curves are shown in Figs. 5(a) and 5(b) for 6‐MV and 15‐MV photon beams, respectively. The PDD data were measured at SSD=100 cm for the depth ranging from 0 cm to 30 cm for field sizes $4\times 4\;{\text{cm}}^{2}$, $10\times 10\;{\text{cm}}^{2}$, and $20\times 20\;{\text{cm}}^{2}$ in the measurement conditions of (1) jaws only (MLC in the park position), (2) MLC only (fixed jaws opening at $35\times 35\;{\text{cm}}^{2}$), and (3) jaws with MLC (zero gap). All data were normalized to the dose at $d_{\text{max}}$ of the field size $10\times 10\;{\text{cm}}^{2}$ generated by the jaws only with the MLC in the park position. This normalization criterion is generally used to measure PDD data at the time of commissioning and acceptance testing of a LINAC. From these curves, it is clear that the surface dose (SD) and the dose in the buildup region are smaller for field sizes defined by jaws with a MLC than that of field sizes defined by jaws only and/or a MLC only. The position of $d_{\text{max}}$ shifted toward the surface more appreciably in 15‐MV photon beams than in 6‐MV photon beams with increasing field size, but does not change with the field‐defining method. No significant difference is seen in the PDD beyond the $d_{\text{max}}$, that is, between the depth of $d_{\text{max}}$ to 30 cm, for both photon energies. The change in PDDs for all three field‐defining methods is within ±0.5% and ±1.0% for 6‐MV and 15‐MV beams, respectively.

### C. Beam profile and penumbra

The beam profiles were measured for the square field sizes of $4\times 4\;{\text{cm}}^{2}$, $10\times 10\;{\text{cm}}^{2}$, $20\times 20\;{\text{cm}}^{2}$, and $30\times 30\;{\text{cm}}^{2}$ in the cross‐plane orientation at the depth of $d_{\text{max}}$ and 10 cm for field sizes defined by (1) jaws only (MLC in the park position), (2) MLC only (fixed jaws opening at $35\times 35\;{\text{cm}}^{2}$), and (3) jaws plus MLC. There was no significant difference in the portions of the beam profiles for more than 80% dose levels. The flatness and symmetry of the beam profiles were also determined for the fields defined by the above methods at $d_{\text{max}}$ and 10‐cm depths. No significant differences were seen among the datasets of flatness and symmetry for all three field size‐defining methods. Similar results were also reported in literature.^(10)^ The widths between 50% dose levels, across the beam profile, at $d_{\text{max}}$ for both 6‐MV and 15‐MV photon beams were computed and are listed in Tables 3and 4, respectively. The width between 50% dose levels represents the field dimension at the depth where it is taken. This width for field sizes defined by MLC only is larger than the width for field sizes defined by jaws only and/or jaws plus MLC, for both 6‐MV and 15‐MV photon beams, and the difference is higher for small field sizes compared to the larger field sizes. The widths between 50% dose level for the field sizes defined by jaws only and/or jaws plus MLC are very similar to each other for both photon energies.

**Table 3:** Width between 50% dose levels at $d_{\text{max}}$ for the 6‐MV photon beam

Field width at SSD=100 cm	Width between 50% dose levels defined by jaws and MLC at $d_{\text{max}}$	Width between 50% dose levels defined by jaws only at $d_{\text{max}}$	Width between 50% dose levels defined by MLC only at $d_{\text{max}}$
40 mm	40.8 mm	40.7 mm	42.7 mm
100 mm	101.3 mm	101.3 mm	103.1 mm
200 mm	203.6 mm	203.7 mm	204.9 mm
300 mm	305.0 mm	305.1 mm	306.0 mm

**Table 4:** Width between 50% dose levels at $d_{\text{max}}$ for the 15‐MV photon beam

Field width at SSD=100 cm	Width between 50% dose levels defined by jaws and MLC at $d_{\text{max}}$	Width between 50% dose levels defined by jaws only at $d_{\text{max}}$	Width between 50% dose levels defined by MLC only at $d_{\text{max}}$
40 mm	41.5 mm	41.3 mm	43.5 mm
100 mm	103.0 mm	103.1 mm	105.2 mm
200 mm	206.7 mm	206.9 mm	208.3 mm
300 mm	309.1 mm	310.1 mm	310.9 mm

**Figure 6:**
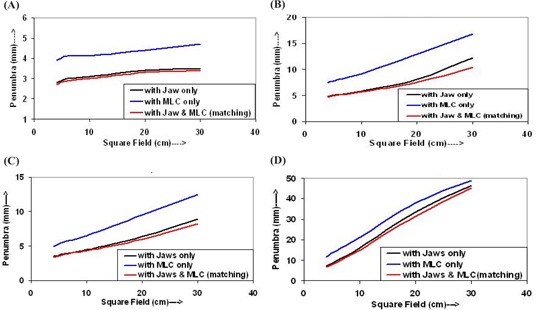
Plot for penumbra width versus field size (a) between 20% and 80% dose levels at $d_{\text{max}}$, (b) between 10% and 90% dose levels at $d_{\text{max}}$, (c) between 20% and 80% dose levels at 10 cm, and (d) between 10% and 90% dose levels at 10 cm for the 6‐MV photon beam

**Figure 7:**
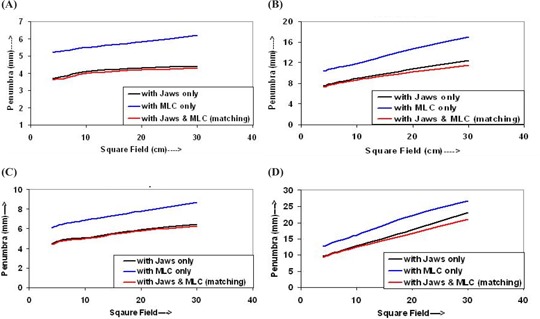
Curves shown for plots of penumbra width versus field size (a) between 20% and 80% dose levels at $d_{\text{max}}$, (b) between 10% and 90% dose levels at $d_{\text{max}}$, (c) between 20% and 80% dose levels at 10 cm, and (d) between 10% and 90%

The penumbra widths were measured at $d_{\text{max}}$ and 10‐cm depths for both 6‐MV and 15‐MV photon beams for the above‐described measurement conditions and field sizes, and are shown in Figs. 6 and 7, respectively. The plots between penumbra widths, for dose levels of 20% to 80% and 10% to 90% at depths of $d_{\text{max}}$ and 10 cm, and field sizes are shown in Figs. 6(a) and 6(b), and in Figs. 6(c) and 6(d) for 6‐MV photon beam, respectively. Figures 7(a) and 7(b), and Figs. 7(c) and 7(d) are the curves for the penumbra widths at depths of $d_{\text{max}}$ and 10 cm, respectively, for the 15‐MV photon beam. Figures 7(a) and 7(c) and Figs. 7(b) and 7(d) show the curves for the penumbra widths for dose levels of 20% to 80% and 10% to 90% dose levels, respectively.

In Figs. 6(a) and 7(a), the penumbra widths of the fields defined by jaws only and/or jaws plus MLC are smaller than those of the field defined by a MLC only. The penumbra curves are parallel to each other and increase slowly with increasing field size. The curves for the fields defined by jaws only have slightly higher values than that of the field defined by jaws plus MLC (matching), for both energies. It is clear from Figs. 6(b), 6(c), and 6(d), and Figs. 7(b), 7(c), and 7(d) that the penumbra widths for the fields defined by MLC only have values larger than those of fields defined by jaws only and/or jaws plus MLC. The penumbra widths for the field defined by jaws only and/or jaws with MLC are indistinguishable from each other for small fields.

## IV. CONCLUSION

The dosimetric characteristics of the Varian Millennium 80 MLC system are quite similar to the standard collimator (jaws) system except for scatter factors, surface dose and buildup region dose, and penumbra widths. For implementation of IMRT, dynamic as well as step‐and‐shoot, these measurements are quite useful.^(^ ^18^ ^–^ ^22^ ^)^

A practical method for calculating scatter factors (i.e., dose rate) for combined fields shaped by jaws and MLC, in the Varian LINACs, has been presented. The method is useful because the equations presented in this study require only scatter factors and reference dose rates. It can also be concluded that the fields defined by a MLC only have penumbra values that are typically 1 mm to 2 mm greater than the penumbra values of the fields defined by jaws only and/or jaws with MLC. Finally, it is suggested that at the time of commissioning of the MLC in the Varian linear accelerators, all the above factors must be determined carefully and must be incorporated into the calculation system.

It appears that the semi‐empirical methods and in manual calculations, for Varian LINACs with a MLC, the contribution of MLC scattering and penumbra should be taken into account for accurate dose and/or monitor unit calculations.
